# Upregulation of *RPLP1* in PBMCs as a screening biomarker for melanoma

**DOI:** 10.1371/journal.pone.0350742

**Published:** 2026-06-16

**Authors:** Yada Wuttithantawee, Achita Wuttithantawee, Rada Wuttithantawee, Pattamaporn Pumpong, Natthamon Sukphanit, Tanamon Chinnakarn, Khunnapat Phuengprajit, Jiraroch Meevassana, Charoenchai Puttipanyalears

**Affiliations:** 1 Faculty of Medicine, Chulalongkorn University, Bangkok, Thailand; 2 Medical Sciences Program, Cell Biology and Human Molecular Genetics, Faculty of Medicine, Chulalongkorn University, Bangkok, Thailand; 3 Faculty of Dentistry, Bangkok Thonburi University, Bangkok, Thailand; 4 Department of Anatomy, Faculty of Medicine, Chulalongkorn University, Bangkok, Thailand; 5 Center of Excellence in Molecular Genetics of Cancer and Human Diseases, Department of Anatomy, Faculty of Medicine, Chulalongkorn University, Bangkok, Thailand; Weill Cornell University, UNITED STATES OF AMERICA

## Abstract

Early detection of melanoma is essential for improving patient outcomes. This research aimed to identify upregulated gene expression in peripheral blood mononuclear cells (PBMCs) induced by secretory factors derived from melanoma cells. These gene alterations may serve as potential non-invasive biomarkers for melanoma detection. Melanoma gene expression profiles from NCBI database were analyzed using the bioinformatics tool (CU-DREAM). A total of 85 participants were enrolled in the study. Expression levels of seven candidate genes were evaluated in a coculture model using PBMCs from healthy participants (n = 5) and melanoma cell lines at 24, 48, and 72 hours by quantitative real-time polymerase chain reaction (qRT-PCR). Based on these findings, the upregulated gene was further examined in PBMC samples for clinical expression analysis, including melanoma patients (n = 15), patients with various cancer types (n = 35), and healthy controls (n = 30). Receiver operating characteristic (ROC) curve analysis was carried out to determine the diagnostic performance of the candidate gene. Among the seven candidate genes, *RPLP1* mRNA expression showed statistically significant upregulation in PBMCs cocultured with both melanoma cell lines, A375 (*p* = 0.0328) and SK-MEL-28 (*p* = 0.0311), at 24 hours compared with PBMC controls. The *RPLP1* expression in PBMC blood samples also showed upregulation in melanoma patients compared to healthy controls (*p* = 0.0006). *RPLP1* upregulation demonstrated good discriminative performance in this cohort with 93.30% sensitivity, 70.00% specificity, and an area under the curve (AUC) of 0.813 (*p* < 0.0001). *RPLP1* upregulation in PBMCs may reflect a cancer-associated signal with relative enrichment in melanoma. Although elevated expression was also observed in breast cancer PBMCs, *RPLP1* may still have potential as a minimally invasive and cost-effective screening biomarker for melanoma, given its high sensitivity in this study cohort. Further validation in larger, independent cohorts is required before clinical application.

## Introduction

Malignant melanoma is a skin cancer arising from the abnormal proliferation of atypical melanocytes. Among skin cancers, melanoma is the most aggressive due to its high metastatic potential and accounts for over 80% of global skin cancer mortality [1]. Early detection of melanoma has been shown to significantly improve morbidity and mortality [2,3]; however, misdiagnosis may occur, resulting in missed early-stage melanoma or unnecessary excision of benign lesions. Dermoscopy is widely used to aid clinical decision-making, but its interpretation remains operator dependent. Knowledge-based errors and technical mistakes are among the most common causes of dermoscopic misdiagnosis [4]. A meta-analysis reported that the sensitivity and specificity of melanoma detection performance were 79.9% and 70.9%, respectively, for general practitioners, and 87.5% and 81.4% for dermatologists [5]. Suspicious melanocytic lesions ultimately require histopathological confirmation, for which excisional biopsy remains the gold standard [6]; however, early biopsy may occasionally lack sufficient histopathological features for definitive diagnosis [7]. These challenges highlight the need for alternative, high-performance, minimally invasive screening tools.

Despite advances in melanoma diagnosis and treatment, reliable blood-based biomarkers remain limited. Serum lactate dehydrogenase (LDH), serum S100B protein, and circulating tumor DNA (ctDNA) assays detecting melanoma-associated mutations such as *BRAF* and *NRAS* have been investigated as promising biomarkers. However, these biomarkers show limited sensitivity in early-stage melanoma and require further large prospective validation [8–11]. Recent studies have found that secretory factors from cancer cells can alter peripheral blood mononuclear cells (PBMCs), including changes in gene expression and epigenetic profiles, which can develop as promising minimally invasive cancer-screening markers [12–17].

This study aimed to examine upregulated gene expression in PBMCs induced by melanoma cell–derived secretory factors as a potential novel tumor marker for melanoma screening. This approach may enable earlier detection of melanoma, potentially before clinical manifestation, thereby improving survival rates. In addition, such biomarkers may assist in clinical decision-making for patients with suspicious melanocytic lesions, particularly in ambiguous cases, by reducing misdiagnosis, avoiding missed early-stage melanoma, and limiting unnecessary invasive excision of benign lesions.

## Materials and methods

### Bioinformatics analysis

Two Gene Expression Omnibus (GEO) datasets from the National Center for Biotechnology Information (NCBI) were obtained, including GSE73333 [18] and GSE114445 [19]. GSE73333 profiles featured gene expression in monocytes cocultured with the A375 melanoma cell line, while GSE114445 represented gene expression profiles of melanoma tissue samples. Relevant literature was retrieved from PubMed (https://pubmed.ncbi.nlm.nih.gov/) using the following terms: (Melanoma) AND (*Homo sapiens*) AND (Peripheral blood mononuclear cell OR PBMC OR White blood cell OR WBC). Both datasets of gene expressions were then analyzed to identify the intersection genes using the Connection Up and Down Regulation Expression Analysis of Microarrays (CU-DREAM) [20]. Functional annotation and gene clustering were performed using DAVID bioinformatics resources [21,22]. Genes within the most significantly enriched clusters were selected for further experimental validation.

### Study participants

The study was conducted from 1 April 2025–15 February 2026 at the Department of Anatomy, Faculty of Medicine, Chulalongkorn University. A total of 85 participants were recruited overall from the Surgery Outpatient Department, King Chulalongkorn Memorial Hospital. Healthy controls (n = 35) were individuals without a personal or family history of cancer or immune-related disorders. Newly diagnosed melanoma (n = 15) and various types of cancer (n = 35), including colorectal cancer (n = 5), cholangiocarcinoma (n = 5), breast cancer (n = 5), thyroid cancer (n = 5), ovarian cancer (n = 5), liver cancer (n = 5), and gastric cancer (n = 5), were enrolled with pathological confirmation. All participants were aged 18 years or older, Asian descent, and provided written informed consent before enrollment. Among the 35 healthy controls, 5 were used for the coculture experiment. The remaining 30 healthy controls were included in the clinical PBMC expression analysis together with 15 melanoma patients and 35 patients with non-melanoma cancers. A participant flow diagram and a detailed summary of sample distribution and usage in each experiment are provided in S1 Fig.

### Sample size calculation

Sample size was calculated using the following formula [14], based on the preliminary results from GSE73333 and GSE114445:

$n=\frac{\left[{\left(Z_{\alpha /\text{2}}+Z_{\beta }\right)}^{\text{2}}\left(\overset{\text{2}}{\underset{d}{\sigma }}\right)\right]}{{\left({\overset{―}{X}}_{d}\right)}^{\text{2}}}$

n  = Sample size

d  = Difference in values between groups.

${\overset{―}{X}}_{d}$ = Mean difference between groups. $\overset{2}{\underset{d}{\sigma }}$ = Variance of the differences between groups.

𝑍𝛼/2 = Standard normal variate for the significance level.

𝑍𝛽 = Standard normal variate for statistical power.

The calculated minimum sample size was 13 (12.99) per group (melanoma and healthy controls), ensuring that the study included an adequate number of samples for downstream analyses.

### Blood sample collection and isolation of peripheral blood mononuclear cells (PBMCs)

Two milliliters of peripheral venous blood were obtained from each participant and collected in EDTA tubes. After dilution with phosphate-buffered saline (PBS) at a 1:1 ratio, peripheral blood was gently layered onto Ficoll-Hypaque density gradient medium (Amersham Pharmacia, Uppsala, Sweden). Samples were centrifuged for 20 min at 1,000 × g at room temperature with the brake off. The PBMC layer formed at the interface between plasma and Ficoll medium was aspirated and washed two times with PBS for 10 min at 650 × g. Purified PBMCs were subsequently used for coculture experiments or RNA extraction.

### Cell culture

The human melanoma cell lines, A375 (*BRAF* V600E, *CDKN2A* mutation, *PTEN* loss) and SK-MEL-28 (*BRAF* V600E, *NRAS* wild type) were obtained from ATCC (Manassas, VA, USA). A375 and SK-MEL-28 were grown in DMEM and EMEM (Gibco, MA, USA), respectively. Culture media were enriched with 10% FBS and 1% penicillin–streptomycin (10,000 U/mL and 10,000 µg/mL) (Gibco, MA, USA) at 37 °C under humidified conditions with 95% air and 5% CO_2_. The cell line was sub-cultured twice a week, and the medium was refreshed every 3 days. The cell line was then harvested with 0.25% Trypsin-EDTA (500 µL) (Gibco, MA, USA), centrifuged for 5 min at 500 × g, and washed with PBS.

### Coculture technique

PBMCs from five healthy donors were cocultured with two types of melanoma cell lines, A375 and SK-MEL-28. The coculture experiment was conducted to allow secretory factors from melanoma cell lines to interact with healthy donor PBMCs. Melanoma cell lines (5 x 10^4^ cells/well) were plated in 24-well culture plates with either DMEM or EMEM enriched with 10% FBS for 24 hours. The PBMCs (1 x 10^5^ cells/well) were then seeded in the polycarbonate membrane Transwell inserts with a pore size of 0.4 µm (Corning, NY, USA), and cocultured for 24, 48, and 72 hours [12,15]. PBMCs were then harvested for RNA extraction and qRT-PCR analysis.

### RNA extraction

PBMC samples were lysed using TRIzol reagent (1 mL) (Thermo Fisher Scientific, MA, USA) and incubated for 5 min at room temperature. Next, chloroform (200 µL) was added, and samples were allowed to incubate for an additional 3 min before centrifugation for 15 min at 12,000 × g at 4 °C to achieve phase separation. The transparent upper aqueous phase was moved to another tube, enriched with RNase-free glycogen (10 µg) and 100% isopropanol (500 µL), incubated for 10 min at room temperature, and then centrifuged for 15 min at 12,000 × g at 4 °C. The supernatants were removed, and RNA pellets were washed with 75% ethanol (1 mL), mixed by vortexing, and centrifuged for 5 min at 7,500 × g at 4 °C. Then, supernatants were removed, and RNA pellets were briefly air-dried before being dissolved in DEPC water (30 µL). RNA concentration was measured using a NanoDrop 2000 spectrophotometer (Thermo Fisher Scientific, DE, USA), and RNA integrity was assessed using an Agilent 2100 Bioanalyzer (Agilent Technologies, CA, USA).

### Complementary DNA (cDNA) synthesis

Following RNA extraction using TRIzol reagent, the cDNA was generated using RevertAid First Strand cDNA Synthesis (Thermo Fisher Scientific, MA, USA). Briefly, all kit components were thawed, mixed, and centrifuged prior to use. The reverse transcription reaction contained template RNA (0.1 ng – 5 µg), primer (1 µL), nuclease-free water (up to 12 µL), 5X reaction buffer (4 µL), RiboLock RNase inhibitor (1 µL), 10 mM dNTP mix (2 µL), and RevertAid Reverse Transcriptase (1 µL). After gentle mixing and centrifugation, the reaction mixtures were incubated for 5 min at 25 °C followed by 60 min at 42 °C. The reaction was subsequently inactivated by heating for 5 min at 70 °C. The resulting first-strand cDNA was then selected for qRT-PCR analysis.

### Primer design

Specific primers of each candidate gene were designed from the NCBI gene expression sequence and then synthesized by BIONICS, Korea. Conventional PCR and electrophoresis were conducted prior to qRT-PCR to optimize the annealing temperatures of each primer (Table 1).

**Table 1:** Primer characteristics used for qRT-PCR analysis of seven candidate genes.

Gene	Forward primer	Reverse primer	Melting temperature (°C)	Product length (base pairs)
RPLP1	5’ CTA CTC GGC CCT CAT TCT GC 3’	5’ GGC TCA ACA TTT ACA CCG GC 3’	59.0	93
KRT15	5’ GGC TCA AGT AGA ATG AGC TG 3’	5’ GCT GAA CTC CTT CAT CTC CTC 3’	56.3	123
ILF3	5’ CCT TGT CTC ACC ACC AAC CT 3’	5’ CCA GAA GCT CCC AAC TAT GA 3’	56.0	103
RBBP6	5’ CAC CTC CAT ACC CCA GAA GA 3’	5’ CTG TAA GGG GGT GAC CTT GA 3’	57.0	103
KHDRBS1	5’ CTG TAT TGG GAA AGG GCT CA 3’	5’ GGG GGT CCA AAG ACT TCA AT 3’	55.8	124
FNBP1L	5’ AAG CCT CAA AAG AGG GTG GT 3’	5’ CAT CAA TGC GCT GCT GTA GT 3’	59.0	104
CSNK1A1	5’ GGG CGT CAC TGT AAT AAG TTA TTC C 3’	5’ GCG ACT CTG CTC AAT ACC AAG ATG T 3’	58.0	100

Detailed characteristics of forward and reverse primer sequences, melting temperatures (°C), and PCR product lengths (base pairs) are shown.

### Quantitative real-time PCR (qRT-PCR)

The qRT-PCR reaction mixture consisted of SYBR green (Bioline) (10 µL), forward and reverse primers (0.8 µL each), cDNA (1 µL), and distilled water (7.4 µL). The reactions were conducted on QuantStudio6 (Thermo Fisher Scientific, MA, USA) in accordance with the manufacturer’s instructions. PCR was conducted under the following conditions: initial denaturation for 2 min at 95 °C, followed by 40 amplification cycles consisting of denaturation for 5 s at 95 °C and annealing/extension for 30 s at 59 °C. At the end of the annealing/extension step, fluorescence emitted from amplified products was detected in real time. All reactions were performed in duplicate. The housekeeping or reference gene was glyceraldehyde 3-phosphate dehydrogenase (*GAPDH*) utilized for testing and analysis parallel to the candidate genes. Cycle threshold (Ct) values were determined using the following equation:


$$\begin{array}{c} \Delta \Delta Ct=\;\Delta Ct_{sample}\;-\;\Delta Ct_{calibrator} \end{array}$$

The results were subsequently expressed as fold changes in candidate gene expression relative to reference samples, quantified according to the 2^-∆∆Ct^ method [23].

### Specificity test of *RPLP1*

To evaluate the specificity of *RPLP1* as a potential biomarker, we assessed its expression in PBMCs derived from 35 patients with various non-melanoma cancers, comprising colorectal cancer (n = 5), cholangiocarcinoma (n = 5), breast cancer (n = 5), thyroid cancer (n = 5), ovarian cancer (n = 5), liver cancer (n = 5) and gastric cancer (n = 5). Relative expression levels of *RPLP1* mRNA were measured using the same qRT-PCR protocol described above. Expression patterns in these groups were compared with those observed in melanoma patients and healthy controls to determine whether *RPLP1* upregulation was specific to melanoma or shared across multiple cancer types.

### Receiver operating characteristic (ROC) analysis and internal validation

ROC curves were analyzed using MedCalc program version 22.009 (Ostend, Belgium) on Windows version 11.3.0.0. Ct values of the candidate gene were entered into the dataset for subsequent analysis. Sensitivity, specificity, and area under the curve (AUC) were computed for a dataset. Moreover, to further assess the robustness of the ROC findings, internal validation was performed using a logistic regression model with stratified three-fold cross-validation to preserve the 2:1 ratio of healthy controls to melanoma cases across folds (S2 Fig).

### Statistical analysis

Bar plots and statistical analyses were generated using GraphPad Prism version 8.0.0 (California, USA). Data are presented as mean ± SD. Intergroup comparisons of gene expression levels were performed using unpaired two-tailed t-tests and Pearson’s correlation analysis. Statistical significance was defined as *p* < 0.05.

### Ethical statement

Ethical approval for this study was granted by the Ethics Committee of the Faculty of Medicine, Chulalongkorn University, Thailand (IRB No. 0864/67). All research procedures were carried out in compliance with the Declaration of Helsinki. Written informed consent was acquired from each participant before inclusion in the study.

## Results

### Bioinformatics data

**Fig 1:**
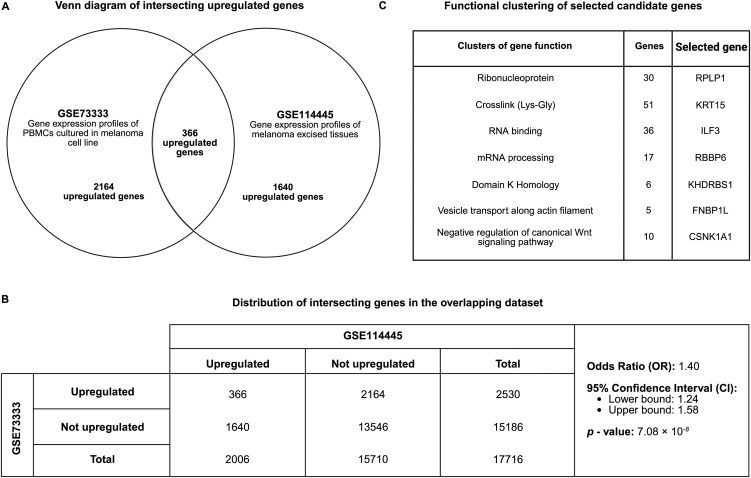
The results of the bioinformatics approach from CU-DREAM program. **(A)** A Venn diagram illustrates the shared upregulated genes between the two datasets (GSE73333 and GSE114445). **(B)** The details of the number of upregulated and not upregulated genes from the intersection of GSE73333 and GSE114445 datasets (OR = 1.40, 95% CI: 1.24–1.58, *p* = 7.08 × 10^*−8*^). **(C)** A table of selected gene function clusters presents a list of seven candidate genes in each cluster including *RPLP1*, *KRT15*, *ILF3*, *RBBP6*, *KHDRBS1*, *FNBP1L*, and *CSNK1A1*.

The results of the CU-DREAM analysis demonstrated a statistically significant directional concordance in differential gene expression between GSE73333 (gene expression profiles in monocytes cocultured with the A375 melanoma cell line), and GSE114445 (gene expression profiles in melanoma tissue samples). Intersection analysis identified 366 upregulated genes (Fig 1A). The details of significant genes within the concordant upregulated and downregulated intersection sets were determined (OR = 1.40, 95% CI: 1.24–1.58, *p* = 7.08 × 10^*−8*^) (Fig 1B). Among the 366 upregulated genes, the functional classification of the genes using the DAVID Bioinformatics resource (https://davidbioinformatics.nih.gov/) found 63 functional clusters. Seven clusters involved in cellular immune signaling with the strongest statistical significance were determined including ribonucleoprotein (30 genes), crosslink: glycyl lysine isopeptide (Lys-Gly) (51 genes), RNA binding (36 genes), mRNA processing (17 genes), domain K homology (6 genes), vesicle transport along actin filament (5 genes), and negative regulation of canonical Wnt signaling pathway (10 genes). Seven candidate genes from the functional cluster were selected based on biological relevance and statistical significance, including *RPLP1* (*p* = 3.56 × 10^*−4*^), *KRT15* (*p* = 6.85 × 10^*−5*^), *ILF3* (*p* = 2.05 × 10^*−7*^), *RBBP6* (*p* = 2.32 × 10^*−4*^), *KHDRBS1* (*p* = 3.12 × 10^*−7*^), *FNBP1L* (*p* = 2.53 × 10^*−4*^), and *CSNK1A1* (*p* = 1.97 × 10^*−8*^) (Fig 1C).

### Identification of upregulated gene expression in coculture model

**Fig 2:**
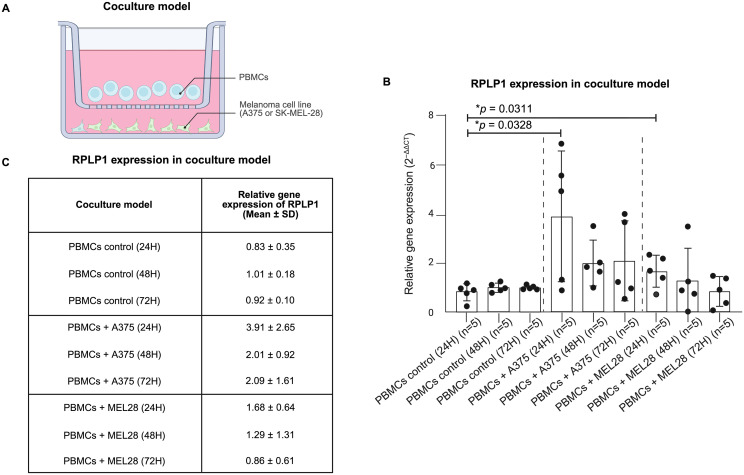
The results of *RPLP1* expression level in coculture model. **(A)** The coculture model demonstrated that secretory factors from melanoma cell lines induced healthy PBMCs through a 0.4 µm pore polycarbonate membrane cell culture insert. **(B)** The relative gene expression (2^-∆∆Ct^) of *RPLP1* gene in PBMCs cocultured with both A375 and SK-MEL-28 cell lines compared with PBMC controls, at 24, 48, and 72 hours, which revealed that *RPLP1* gene showed a statistically significant increase in expression in PBMCs cocultured with both A375 (p = 0.0328) and SK-MEL-28 (p = 0.0311), compared with PBMC controls, at 24 hours. **(C)** The level of *RPLP1* expression in PBMC controls, PBMCs with A375 and PBMCs with SK-MEL-28, at 24, 48, and 72 hours, was represented by mean ± SD.

Relative gene expression profiles (2^-∆∆Ct^) of the seven candidate genes were analyzed in 5 healthy control PBMCs cocultured with two types of melanoma cell lines (A375 and SK-MEL-28), at 24, 48, and 72 hours. The illustration of the coculture model between the melanoma cell line and PBMCs is shown in Fig 2A. Among the seven genes, we found that only *RPLP1* exhibited an upregulated expression level in PBMCs following coculture with melanoma cell lines compared with PBMC controls. The *RPLP1* expression showed a statistically significant increase in PBMCs cocultured with A375 (*p =* 0.0328) and SK-MEL-28 (*p* = 0.0311), in comparison with PBMC controls, at 24 hours and declined at later time points (Fig 2B). The relative gene expression levels of *RPLP1* (mean ± SD), including PBMC controls, PBMCs cocultured with A375, and PBMCs cocultured with SK-MEL-28 at 24, 48, and 72 hours, are presented in Fig 2C. In the PBMC control group, the relative mRNA expression of *RPLP1* was 0.83 ± 0.35, 1.01 ± 0.18 and 0.92 ± 0.10 at 24, 48, and 72 hours, respectively. In the PBMCs cocultured with A375 cell group, the expression level was 3.91 ± 2.65, 2.01 ± 0.92 and 2.09 ± 1.61 at corresponding time points. In the PBMCs cocultured with SK-MEL-28 cell group, the relative mRNA expression was 1.68 ± 0.64, 1.29 ± 1.31 and 0.86 ± 0.61 at 24, 48, and 72 hours, respectively. In contrast, the *FNBP1L* gene was downregulated in PBMCs cocultured with A375 at 24 hours (*p* = 0.0385). No other genes exhibited statistically significant changes in expression (S3 Fig).

### *RPLP1* expression in PBMCs from melanoma patients and healthy controls

**Fig 3:**
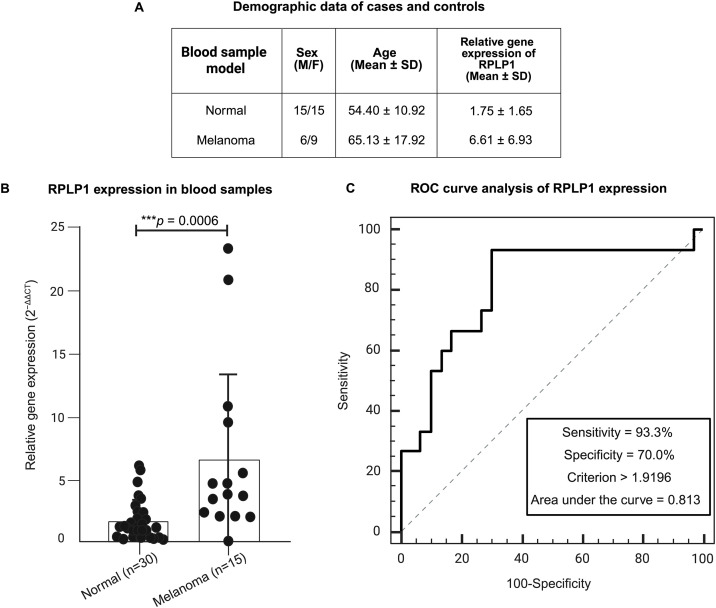
The results of *RPLP1* expression levels in blood sample model. **(A)** The demographic data, representing sex, age and relative gene expression of *RPLP1* between melanoma patients and healthy controls (Mean ± SD). **(B)** The comparison of *RPLP1* expression levels between melanoma patients (n = 15) and healthy controls (n = 30) was statistically significant (*p* = 0.0006), and **(C)** the ROC curve analysis of *RPLP1* expression shows an AUC of 0.813 (95% CI: 0.669-0.914, *p* < 0.0001) with 93.30% sensitivity and 70.00% specificity.

The gene expression profile of *RPLP1* was examined in PBMCs derived from melanoma patients (n = 15) across all clinical stages and from healthy controls (n = 30). The healthy control group showed a male-to-female ratio of 1:1 with a mean age of 54.40 ± 10.92 years, while the melanoma group showed a male-to-female ratio of 2:3 and an average age of 65.13 ± 17.92 years as shown in the demographic data (Fig 3A). Pearson’s correlation analysis showed no significant association between age and *RPLP1* expression in either the healthy control group (r = −0.074, *p* = 0.696) or the melanoma group (r = −0.364, *p* = 0.183) (S4 Fig).

Quantitative real-time PCR (qRT-PCR) was carried out for analysis, and relative expression levels compared to the internal control gene were quantified according to the 2^-∆∆Ct^ method. The melting temperature for *RPLP1* was 59 °C, and *GAPDH* was employed as the internal reference gene. No apparent difference in *GAPDH* Ct values was observed between melanoma patients and healthy controls, supporting its use for normalization (S5 Fig). Interestingly, the relative expression level of *RPLP1* in the melanoma PBMCs (6.61 ± 6.93) was significantly increased when compared to healthy control PBMCs (1.75 ± 1.65) (*p* = 0.0006) (Fig 3B).

### ROC analysis and internal validation

The diagnostic performance of *RPLP1* was evaluated using ROC curve analysis. *RPLP1* expression demonstrated good diagnostic performance, with AUC of 0.813 (95% CI: 0.669–0.914, *p* < 0.0001), 93.30% sensitivity, and 70.00% specificity (Fig 3C). To further assess the robustness of the observed ROC performance, internal validation was performed using a logistic regression model with stratified three-fold cross-validation. The validation sets demonstrated consistent exploratory discriminatory performance, with AUC values of 0.780, 0.860, and 0.800, across the three folds, corresponding to a mean cross-validated AUC of 0.813 (S2 Fig).

### Specificity of *RPLP1* upregulation in melanoma

**Fig 4:**
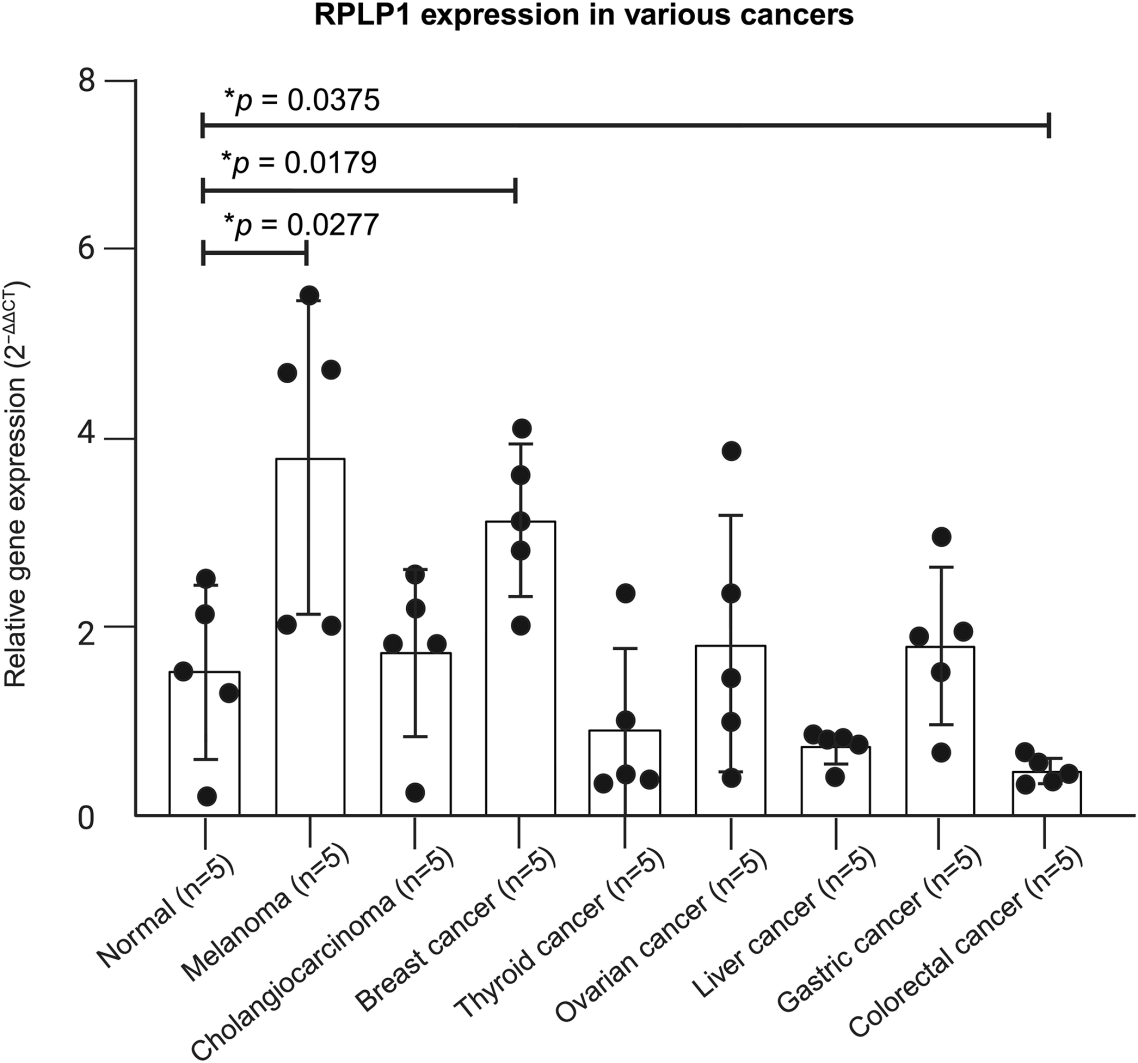
Comparison of RPLP1 gene expression across melanoma and non-melanoma cancer groups. The mRNA expression level of *RPLP1* gene was significantly increased in PBMCs from melanoma patients (*p* = 0.0277) and breast cancer patients (*p* = 0.0179), compared to healthy controls, but the mRNA expression level of *RPLP1* gene of colorectal cancer was significantly decreased (*p* = 0.0375).

To further assess the specificity of *RPLP1*, the relative gene expression of *RPLP1* was observed in PBMCs of various cancers, including colorectal cancer (n = 5), cholangiocarcinoma (n = 5), breast cancer (n = 5), thyroid cancer (n = 5), ovarian cancer (n = 5), liver cancer (n = 5) and gastric cancer (n = 5). The healthy control (n = 5) and melanoma (n = 5) samples were randomly selected using simple random sampling from the clinical PBMC cohort described above. This subset analysis was performed to provide comparison groups of equal size to each non-melanoma cancer group (n = 5 per cancer type). *RPLP1* gene expression was shown to be upregulated in PBMCs from blood samples of melanoma (*p* = 0.0277) and breast cancer (*p* = 0.0179), compared with normal PBMCs. In contrast, downregulation of *RPLP1* was found in PBMCs of colorectal cancer (*p* = 0.0375) compared with normal PBMCs. The *RPLP1* expression in PBMCs of cholangiocarcinoma, thyroid cancer, ovarian cancer, liver cancer, and gastric cancer showed no significant differences from normal PBMCs (Fig 4).

## Discussion

In this study, we identified *RPLP1* as an upregulated gene in PBMCs of melanoma patients. *RPLP1* mRNA expression was significantly higher in melanoma patients compared with healthy controls (*p* = 0.0006). ROC analysis demonstrated good discriminatory performance in this study cohort with 93.30% sensitivity, 70.00% specificity, and an AUC of 0.813 (95% CI: 0.669–0.914, *p* < 0.0001). Moreover, internal cross-validation showed consistent performance, yielding AUCs of 0.780, 0.860, and 0.800, across the validation folds, with a mean cross-validated AUC of 0.813. These findings suggest that melanoma is associated with systemic transcriptional alterations in circulating immune cells, potentially reflecting systemic effects of tumor-derived signaling beyond the local tumor microenvironment.

*RPLP1* (ribosomal protein lateral stalk subunit P1) is a structural component of the 60S ribosomal subunit, forming part of the ribosomal P-stalk complex together with RPLP0 and RPLP2. This complex interacts with elongation factors during translation, promoting efficient protein synthesis. Although traditionally considered a housekeeping ribosomal protein, recent studies indicate that its expression can be dynamically regulated in cancer tissue samples [24–33]. Beyond its role in maintaining general protein synthesis, *RPLP1* overexpression has been linked to enhanced cell proliferation, migration, invasion, and poor clinical outcomes in multiple malignancies. These observations suggest that *RPLP1* may function not only as a core ribosomal component but also as a contributor to tumor progression, highlighting its potential as a biomarker and a target for further investigation.

Accumulating evidence suggests that *RPLP1* is upregulated in various cancers [24–33]. To sustain their rapid growth and elevated metabolic activity, cancer cells often increase the expression of ribosomal proteins to meet the greater demand for protein synthesis [32]. Analysis of the GSE114445 dataset further revealed that *RPLP1* is upregulated in melanoma tissue samples when compared to melanocytic nevi and dysplastic nevi tissue samples, by our bioinformatic analysis process through CU-DREAM program [19]. Moreover, several studies have linked *RPLP1* expression to oncogenic pathways in various cancers. The increase of *RPLP1* in triple-negative breast cancer and osteosarcoma was correlated with recurrence, metastasis, and poor survival [24,25,33] by enhancing cell proliferation and migration [26–28,31]. In cervical cancer, *RPLP1* has also been found to activate downstream of *CNN3* (calponin 3) to rescue proliferation and migration. These findings demonstrate that *RPLP1* may function in oncogenic pathways beyond general ribosome activity in cervical cancer [27]. Likewise, *RPLP1* knockdown can downregulate proliferation, migration, and invasion in hepatocellular carcinoma and endometrial adenocarcinoma, supporting its role as a tumor-promoting factor [28,29]. However, *RPLP1* expression is not uniformly regulated across all cancer types. In colorectal cancer, both upregulation and downregulation of *RPLP1* have been reported [30,34], suggesting that its expression may vary across tumor types or experimental context.

**Fig 5:**
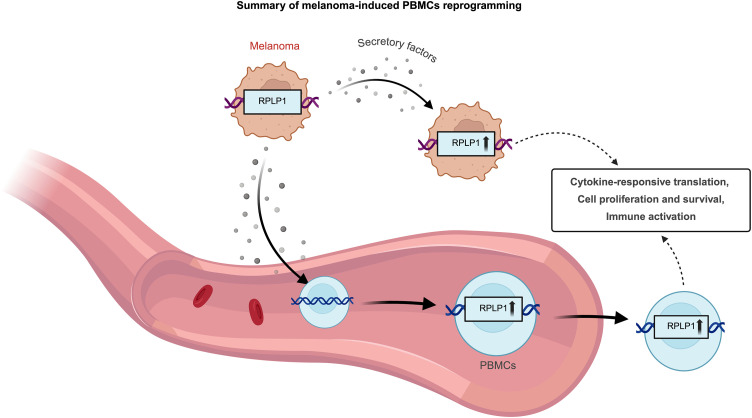
The summarized illustration of melanoma-induced PBMC reprogramming. Melanoma cells release cytokines, chemokines, and extracellular vesicles that influence circulating immune cells, leading to upregulation of *RPLP1* expression. This systemic induction may parallel tumor-intrinsic functions of *RPLP1*, including cytokine-responsive translation, cell proliferation and survival, and immune activation, reflecting a melanoma-associated systemic immune response.

Our study extends these tumor-intrinsic observations to the systemic immune compartment by demonstrating *RPLP1* upregulation in PBMCs, as summarized in Fig 5. Tumor-associated cytokines and inflammatory cues can reprogram circulating immune cells [35–38], and elevated *RPLP1* expression in PBMCs likely reflects immune adaptation to the tumor microenvironment. This systemic induction may parallel the tumor-intrinsic functions of *RPLP1*, such as cytokine-responsive translation, cell proliferation and survival, and immune activation, and may support its utility as a potential peripheral blood biomarker for melanoma. Recent studies provide biological plausibility for our interpretation by demonstrating that *RPLP1* expression can regulate CD8^+^ T cell infiltration in cancerous tissues and control the inflammatory process in activated macrophages [32,39–43]. *RPLP1* also acts as cytokine-driven translational reprogramming during antitumor immune responses [41–43]. Collectively, these findings suggest that tumor-derived secretory factors induce systemic transcriptional responses in circulating immune cells, resulting in measurable gene expression changes in PBMCs that may serve as potential blood-based biomarkers.

We further observed that *RPLP1* expression in PBMCs exhibited variation across different cancer types. While we observed upregulation in PBMCs from melanoma patients, a similar pattern was observed in PBMCs from breast cancer patients, which is consistent with previous studies reporting *RPLP1* upregulation in breast cancer tissues, suggesting that certain tumor types may induce systemic immune responses involving *RPLP1*. In contrast, we found that PBMCs from colorectal cancer patients showed downregulation of *RPLP1*, indicating that the direction of *RPLP1* regulation may depend on tumor biology and tissue context. Given that both upregulation and downregulation of *RPLP1* have been reported in colorectal cancer [30,34,44], our findings further support the notion that *RPLP1* regulation is context-dependent rather than unidirectional. This discrepancy may be explained by differences between tumor tissue-derived expression and PBMC-based measurements, as our study reflects systemic immune responses rather than tumor-intrinsic gene expression. This variability highlights the importance of future studies aimed at clarifying the mechanistic links between tumor-secreted factors and PBMC gene expression.

The transient nature of *RPLP1* induction in coculture may reflect the limitations of the in vitro system. In this setting, the same PBMC population is repeatedly exposed to tumor-derived cytokines and other soluble mediators, which may reduce responsiveness over time. In contrast, circulating PBMCs are continuously renewed in vivo and are exposed to signals from the dynamic tumor microenvironment, allowing newly circulating immune cells to be exposed to tumor-derived factors. This difference may help explain why *RPLP1* induction was transient in coculture but remained elevated in patient PBMCs. In previous work, *STAU2* expression peaked at 48 hours [12], whereas *RPLP1* peaked at 24 hours in the present study, suggesting that temporal responses to tumor-derived signaling may vary by gene. Despite the limited number of healthy donors, the observed effect was seen in coculture with both melanoma cell lines, supporting biological reproducibility.

Importantly, the absence of stage-stratified analysis represents a significant limitation of the present study. This was primarily due to the relatively small number of melanoma cases, which precluded reliable subgroup analysis in this cohort. Although some PBMC-based biomarker studies have reported that tumor-induced transcriptional responses in circulating immune cells may not strongly depend on tumor stage, these findings should be interpreted cautiously and may not be generalizable across different tumor types or experimental settings. For example, *STAU2* expression showed minimal variation across breast cancer stages, whereas *FLNA*, *CLU*, and *p16*^*INK*^*^4A^* expression were not reported to differ significantly according to disease stage or tumor burden in other cancer types [12,14,17]. These observations suggest that tumor-derived secretory factors can induce systemic immune responses even at relatively low tumor burden. Nevertheless, the relevance of *RPLP1* expression across different melanoma stages remains to be clarified in larger cohorts with adequate stage representation.

Several other limitations should be considered. The study was conducted with a limited sample size and a cross-sectional, single-center design, which may restrict the generalizability of the findings. In addition, candidate gene selection was based on a monocyte-derived dataset, whereas validation was performed in whole PBMCs, which may have influenced gene expression profiles and candidate selection, partly due to the limited availability of suitable public datasets. The tumor specificity of *RPLP1* is also limited. Although *RPLP1* was upregulated in PBMCs from patients with melanoma, elevated expression was also observed in PBMCs from patients with breast cancer in our cohort, indicating that its expression is not restricted to melanoma. This limited tumor specificity may increase the risk of false-positive results if *RPLP1* is used in a clinical screening context. Therefore, *RPLP1* may represent a cancer-associated signal with relative enrichment in melanoma rather than a melanoma-specific biomarker. The mechanistic basis of *RPLP1* upregulation was not directly investigated, and the findings should therefore be considered hypothesis-generating. Moreover, PBMC heterogeneity, the lack of cell subtype-specific analysis, and the absence of treatment-response evaluation may also have introduced biological and clinical variability, warranting further functional and cell-specific studies. Overall, these findings should be regarded as preliminary and require validation in larger, independent, well-characterized cohorts with stage stratification, treatment-response assessment, and external validation.

Although *RPLP1* is primarily known as a ribosomal protein, its elevated expression in PBMCs suggests potential relevance in melanoma detection. This may be clinically meaningful because melanoma prognosis is strongly stage-dependent, with five-year survival exceeding 98% in localized disease but decreasing to approximately 22% in patients with distant metastasis [38]. In this context, the high sensitivity observed in our study supports the potential utility of *RPLP1* as a blood-based screening marker, although its limited tumor specificity and the exploratory nature of the present findings should be considered.

## Conclusion

Our study demonstrated that *RPLP1* mRNA was significantly upregulated in PBMCs from melanoma patients, reflecting tumor-induced changes in circulating immune cells. *RPLP1* expression showed good diagnostic performance, with 93.30% sensitivity and 70.00% specificity for distinguishing melanoma patients from healthy controls. Although elevated expression was also observed in PBMCs from patients with breast cancer, *RPLP1* may represent a cancer-associated signal with relative enrichment in melanoma and still hold potential as a minimally invasive screening biomarker in this study cohort. Further validation in larger, independent, multicenter cohorts is required before clinical application.

## Supporting information

S1 Fig Participant flow diagram and sample distribution across experiments. (A) Participant flow diagram showing recruitment and grouping of participants (n = 85). (B) Summary of sample distribution and usage in each experiment.
(TIF)

S2 Fig Internal validation of RPLP1 classification performance using stratified three-fold cross-validation. (A) Schematic representation of the cross-validation design. Each training set contained 20 healthy controls and 10 melanoma patients, and each validation set contained 10 healthy controls and 5 melanoma patients, preserving the original 2:1 class ratio across folds. (B) Receiver operating characteristic (ROC) curves for the validation sets across the three folds, with AUC values of 0.780, 0.860, and 0.800.
(TIF)

S3 Fig The relative gene expression of six candidate genes. The relative gene expression of six candidate genes was compared to the housekeeping gene (*GAPDH*) in PBMCs with both A375 and SK-MEL-28 cell lines compared with PBMC controls, at 24, 48, and 72 hours in coculture model. (A) expression level of *FNBP1L* gene was found to be downregulated only in PBMCs with A375 at 24 hours (*p* = 0.0385). However, the expression level of (B) *ILF3*, (C) *KHDRBS1*, (D) *KRT15*, (E) *RBBP6*, (F) *CSNK1A1* in PBMCs cocultured with melanoma cells compared with the control showed no significant difference.
(TIF)

S4 Fig Correlation between age and PBMCs RPLP1 expression in healthy controls and melanoma patients. Scatter plots showing the relationship between age and relative RPLP1 expression in PBMCs from healthy controls (n = 30) and melanoma patients (n = 15). Correlation was assessed using Pearson’s correlation analysis. No significant association between age and RPLP1 expression was observed in (A) healthy controls (r = −0.074, *p* = 0.696) and in (B) melanoma patients (r = −0.364, *p* = 0.183).
(TIF)

S5 Fig *GAPDH* Ct values in PBMCs from melanoma patients and healthy controls. *GAPDH* Ct values measured by qRT-PCR in PBMCs from melanoma patients and healthy controls. No apparent difference in *GAPDH* Ct values was observed between groups, supporting its use as an internal reference gene for normalization.
(TIF)

S1 File Step-by-step laboratory protocols. The protocols described in this study are available at DOI: https://dx.doi.org/10.17504/protocols.io.q26g7oj78vwz/v1. (Private link for reviewers: https://www.protocols.io/private/DEE10713371311F18A1F0A58A9FEAC02 to be removed before publication).
(PDF)

S2 File Raw data of each experiment. (PDF)
