# Supplementary material for: Upregulation of RPLP1 in PBMCs as a screening biomarker for melanoma
Source: PLoS One. 2026 Jun 16;21(6):e0350742. doi: 10.1371/journal.pone.0350742 (PMC13271437; doi:10.1371/journal.pone.0350742)
Supplement: S1 File — The protocols described in this study are available at DOI: https://dx.doi.org/10.17504/protocols.io.q26g7oj78vwz/v1. (Private link for reviewers: https://www.protocols.io/private/DEE10713371311F18A1F0A58A9FEAC02 to be removed before publication). (PDF) [file pone.0350742.s006.pdf]

Upregulation of RPLP1 in PBMCs as a screening biomarker for melanoma

Yada Wuttithantawee, Achita Wuttithantawee, Rada Wuttithantawee, Pattamaporn Pumpong, Natthamon Sukphanit, Tanamon Chinnakarn, Khunnapat Phuengprajit, Jiraroch Meevassana, Charoenchai Puttipanyalears

Isolation of Peripheral Blood Mononuclear Cells (PBMCs)

1 Prepare PBS and Ficoll-Hypaque density gradient medium at room temperature.

2 Dilute whole blood with PBS at a 1:1 ratio.

3 Carefully layer the diluted blood over Ficoll-Hypaque in a centrifuge tube without disturbing the interface.

4 Centrifuge at 1,000 × g for 20 min at room temperature with the brake off.

5 After centrifugation, identify the PBMC layer at the plasma–Ficoll interface.

6 Carefully aspirate the PBMC layer and transfer it to a new centrifuge tube.

7 Add PBS to the collected PBMCs and mix gently.

8 Centrifuge at 650 × g for 10 min.

9 Discard the supernatant without disturbing the cell pellet.

10 Resuspend the cell pellet in PBS.

11 Repeat the wash step one more time by centrifuging at 650 × g for 10 min.

12 Remove the supernatant and collect the purified PBMC pellet.

13 Use the purified PBMCs immediately for coculture experiments or proceed to RNA extraction.

RNA extraction

14 Lyse the PBMC pellet in 1 mL of TRIzol reagent and incubate the sample at room temperature for 5 min to allow complete dissociation of nucleoprotein complexes.

15 Add 0.2 mL of chloroform per 1 mL of TRIzol reagent to each tube, mix thoroughly, and leave the mixture at room temperature for 3 min.

16 Centrifuge the samples at 12,000 × g for 15 min at 4 °C to separate the mixture into aqueous and organic phases.

17 Carefully transfer the clear upper aqueous phase to a new tube without disturbing the interphase.

18 Add 10 µL of RNase-free glycogen and 500 µL of 100% isopropanol to the aqueous phase, then mix gently.

19 Incubate the mixture at room temperature for 10 min to precipitate the RNA.

20 Centrifuge the samples at 12,000 × g for 15 min at 4 °C to pellet the RNA.

21 Discard the supernatant and wash the RNA pellet with 1 mL of 75% ethanol.

22 Vortex the tube briefly to resuspend the pellet, then centrifuge at 7,500 × g for 5 min at 4 °C.

23 Remove the ethanol completely and air-dry the RNA pellet for 10 min.

24 Dissolve the RNA pellet in 30 µL of DEPC-treated water.

25 Determine RNA concentration and assess RNA integrity using a NanoDrop spectrophotometer and Bioanalyzer.

cDNA Synthesis

26 In a sterile nuclease-free tube placed on ice, combine the RNA template and primer in the appropriate amount. The RNA input may range from 0.1 ng to 5 µg. Primers include 1 µg oligo(dT)18. Add DEPC water to a final volume of 12 µL.

27 If the RNA template is GC-rich or is expected to contain secondary structures, gently mix the reaction, centrifuge briefly, incubate at 65 °C for 5 min, then chill on ice, centrifuge again briefly, and keep on ice.

28 Add the following reagents in order: 4 µL of 5X reaction buffer, 1 µL of RiboLock RNase inhibitor, 2 µL of 10 mM dNTP mix, and 1 µL of RevertAid Reverse Transcriptase, giving a final reaction volume of 20 µL. Mix gently and centrifuge briefly.

29 Incubate first at 25 °C for 5 min, followed by 42 °C for 60 min.

30 Stop the reaction by heating at 70 °C for 5 min. Heat inactivation should be avoided before analysis of long cDNA products to reduce the risk of cleavage.

31 The synthesized cDNA can be used directly for PCR or stored at −20 °C until needed. Typically, 2 µL of the reverse transcription product is sufficient for a 50 µL PCR reaction.

Quantitative real-time PCR (qRT-PCR)

32 Thaw the SYBR Green master mix (Bioline), forward and reverse primers, cDNA template, and distilled water completely. Mix gently and keep all reagents on ice before use.

33 Prepare the qPCR reaction mixture in a total volume of 20 µL for each sample.

34 For each reaction, combine the following components in a PCR tube or plate well: 10 µL SYBR Green master mix, 0.8 µL forward primer, 0.8 µL reverse primer, 1 µL cDNA template, 7.4 µL distilled water.

35 Mix the reaction gently and centrifuge briefly to collect the contents at the bottom of the tube or well.

36 Load the reactions into the QuantStudio 6 Real-Time PCR System (Thermo Fisher Scientific).

37 Perform PCR amplification using the following cycling conditions:
initial denaturation at 95 °C for 2 min
followed by 40 cycles of:
denaturation at 95 °C for 5 s
annealing/extension at 59 °C for 30 s

38 Measure fluorescence at the end of each annealing/extension step in real time.

39 Perform all reactions in duplicate.

40 Run the reference gene GAPDH in parallel with the candidate genes for normalization.

41 Determine the Ct values for each reaction and calculate relative gene expression using the following equations:
$\Delta Ct = Ct\_target - Ct\_reference$
$\Delta\Delta Ct = \Delta Ct\_sample - \Delta Ct\_calibrator$
Relative expression = $2^{-\Delta\Delta Ct}$

Coculture protocol

42 Maintain A375 and SK-MEL-28 melanoma cells in complete culture medium until ready for coculture.

43 Seed melanoma cells into 24-well culture plates at a density of 5×10^4 cells/well in the appropriate complete medium:
A375 in DMEM
SK-MEL-28 in EMEM
Both media should be supplemented with 10% FBS.

44 Incubate the melanoma cells for 24 h at 37 °C in 5% CO_2 to allow cell attachment.

45 Isolate PBMCs from healthy donors and prepare them in culture medium immediately before coculture.

46 Place polycarbonate membrane Transwell inserts (0.4 µm pore size) into each well. The 0.4 µm membrane allows exchange of soluble factors while preventing direct contact between PBMCs and melanoma cells.

47 Seed PBMCs into the upper Transwell insert at a density of 1×10^5 cells/well.

48 Maintain the coculture system at 37 °C under humidified conditions with 5% CO_2.

49 Coculture PBMCs with melanoma cells for 24, 48, and 72 h.

50 At each time point, collect PBMCs from the Transwell inserts.

51 Proceed with RNA extraction followed by qRT-PCR analysis.
