# Supplementary material for: Upregulation of RPLP1 in PBMCs as a screening biomarker for melanoma
Source: PLoS One. 2026 Jun 16;21(6):e0350742. doi: 10.1371/journal.pone.0350742 (PMC13271437; doi:10.1371/journal.pone.0350742)
Supplement: S2 File — (PDF) [file pone.0350742.s007.pdf]

Blood Sample Model: normal

Sample	Sex	Age	RPLP1
Normal 1	M	66	5.77734621
Normal 2	F	60	2.97649034
Normal 3	M	54	0.34948469
Normal 4	F	48	0.22231174
Normal 5	M	38	3.49064466
Normal 6	M	70	4.86666743
Normal 7	F	48	0.18936921
Normal 8	M	45	1.17044758
Normal 9	F	67	0.33321605
Normal 10	F	66	0.5965571
Normal 11	F	65	2.55623606
Normal 12	F	78	1.09503549
Normal 13	F	72	0.96395308
Normal 14	M	54	0.281
Normal 15	F	52	1.21734584
Normal 16	M	49	0.39322973
Normal 17	M	50	1.15332347
Normal 18	M	41	3.7113374
Normal 19	M	48	1.79280952
Normal 20	M	51	0.35903153
Normal 21	F	50	2.42338288
Normal 22	M	44	0.06364886
Normal 23	F	42	1.11099816
Normal 24	F	47	1.55663489
Normal 25	F	61	1.91959582
Normal 26	F	53	0.35588202
Normal 27	M	59	1.89923933
Normal 28	M	61	2.27391718
Normal 29	F	31	6.1372195
Normal 30	M	62	1.12370284

Sex	M15	
	F15	
Age	Mean	54.4
	SD	10.9216646
RPLP1	Mean	1.74533529
	SD	1.64675093

Blood Sample Model: melanoma

Sample	Sex	Age	RPLP1
Melanoma 1	M	51	4.72421347
Melanoma 2	M	74	2.01231719
Melanoma 3	F	76	4.67598799
Melanoma 4	F	81	1.99619605
Melanoma 5	M	43	5.51672427
Melanoma 6	F	66	3.40059933
Melanoma 7	M	47	3.85033623
Melanoma 8	F	69	2.02619106
Melanoma 9	F	68	9.61921212
Melanoma 10	M	77	3.62568952
Melanoma 11	F	75	0.1270609
Melanoma 12	F	96	2.39661434
Melanoma 13	M	35	10.8286616
Melanoma 14	F	39	23.4702773
Melanoma 15	F	80	20.8254219

Sex	M6	
	F9	
Age	Mean	65.1333333
	SD	17.9159413
RPLP1	Mean	6.60636689
	SD	6.92681996

Coculture Model: RPLP1

	PBMCs control (24H)	PBMCs control (48H)	PBMCs control (72H)
	0.895373416	1.1168525	1.006112958
	0.996348945	0.8953734	0.945861173
	0.796125553	0.9963489	0.896237226
	0.259040931	1.2606861	0.759863516
	1.181263869	0.7961256	0.988694735
Mean	0.825630543	1.0130773	0.919353921
SD	0.347267273	0.182538693	0.098754094

	PBMCs + A375 (24H)	PBMCs + A375 (48H)	PBMCs + A375 (72H)
	6.854454766	2.0429737	1.247471968
	4.923379405	1.0069753	0.565799702
	5.556862347	1.848294	0.982056352
	0.899390041	3.502735	3.668915036
	1.320486232	1.6536143	4.005044685
Mean	3.910914558	2.01091846	2.093857549
SD	2.6541598	0.920401947	1.614068895

	PBMCs + MEL28 (24H)	PBMCs + MEL28 (48H)	PBMCs + MEL28 (72H)
	0.744555872	1.252526	1.494150513
	1.414039318	3.4988827	0.09907953
	1.697172987	0.0492312	0.389790985
	2.190057375	0.7609065	0.927245002
	2.346744878	0.8965648	1.388220604
Mean	1.678514086	1.29162224	0.859697327
SD	0.642413794	1.309082916	0.609463246

Coculture Model: FNBP1L

	PBMCs control (24H)	PBMCs control (48H)	PBMCs control (72H)
	2.496432827	1.407731	0.319029
	0.744898212	1.939703	3.134508
	0.535571691	3.134508	1.149766066
	0.971862526	2.496432827	1.258934782
	1.033143705	0.862134	0.690855597
Mean	1.156381792	1.968101765	1.310618689
SD	0.774547558	0.891294536	1.086562632

	PBMCs + A375 (24H)	PBMCs + A375 (48H)	PBMCs + A375 (72H)
	0.26930184	2.696647	0.836076
	0.312579319	0.933623	0.014138585
	0.207371763	1.779400196	3.521032914
	0.243841402	3.294233294	2.63654725
	0.437120731	3.938339226	0.074577919
Mean	0.294043011	2.528448543	1.416474533
SD	0.088683512	1.194471712	1.582864374

	PBMCs + MEL28 (24H)	PBMCs + MEL28 (48H)	PBMCs + MEL28 (72H)
	0.798566083	0.100179042	2.804687219
	0.310608557	1.147581	0.703445708
	0.297591601	4.47261774	0.885841298
	0.639468493	0.012821038	0.000189768
	0.985827623	0.004708152	1.098541
Mean	0.606412471	1.147581394	1.098540999
SD	0.302010701	1.920075435	1.039068309

Coculture Model: KHDRBS1

	PBMCs control (24H)	PBMCs control (48H)	PBMCs control (72H)
	0.725299	1.458645	1.091972
	0.685568	0.564424	0.624996
	0.7650307	0.4494112	0.607221
	0.6670118	0.6794371	0.673224
	1.959691	0.3880771	1.173884
Mean	0.9605201	0.70799888	0.8342594
SD	0.559828844	0.434199451	0.275241103

	PBMCs + A375 (24H)	PBMCs + A375 (48H)	PBMCs + A375 (72H)
	2.111851	1.28941	0.74233
	2.053936	1.039594	0.660425
	0.4873548	0.1859224	0.126164463
	0.3634951	0.3587097	0.340952044
	0.5338014	0.498205	0.879771133
Mean	1.11008766	0.67436822	0.549928528
SD	0.890460835	0.469286523	0.308735767

	PBMCs + MEL28 (24H)	PBMCs + MEL28 (48H)	PBMCs + MEL28 (72H)
	2.5141677	0.1522592	0.526173431
	0.34447	1.4506019	0.559502
	0.1801214	0.211916	0.356349768
	0.5088194	0.3901073	0.614914657
	0.7374009	0.2332465	0.740570773
Mean	0.85699588	0.48762618	0.559502126
SD	0.949024512	0.545442093	0.139855359

Coculture Model: KRT15

	PBMCs control (24H)	PBMCs control (48H)	PBMCs control (72H)
	1.036672626	0.735474	0.434276
	1.32800112	1.815341	2.302681
	0.692220471	1.120293	1.548366331
	0.711568991	1.437886	2.16420369
	0.950526167	0.624473	0.298420153
Mean	0.943797875	1.1466934	1.349589435
SD	0.261488321	0.493557308	0.942635629

	PBMCs + A375 (24H)	PBMCs + A375 (48H)	PBMCs + A375 (72H)
	0.970801	0.861873	1.285145
	2.317813	1.05691	1.313532
	3.140240581	0.236596989	2.528412829
	2.302911206	1.876570148	1.545468371
	0.483008648	0.554441433	0.366843342
Mean	1.842954887	0.917278314	1.407880308
SD	1.087440388	0.620037347	0.771726495

	PBMCs + MEL28 (24H)	PBMCs + MEL28 (48H)	PBMCs + MEL28 (72H)
	1.451505817	0.668752116	1.179868
	1.564269535	0.785213	3.634222256
	0.535594491	0.119728044	0.828426181
	1.214813964	1.603489074	0.087151286
	1.526605346	0.748884122	0.169671809
Mean	1.258557831	0.785213271	1.179867906
SD	0.426358874	0.530878725	1.445846839

Coculture Model: RBBP6

	PBMCs control (24H)	PBMCs control (48H)	PBMCs control (72H)
	0.587970846	1.486496	0.672723
	0.672723	0.523761073	1.486496
	1.486496	0.113595154	1.033033893
	0.724127422	0.672723	0.348085932
	0.904112738	1.404322482	0.700021415
Mean	0.875086001	0.840179542	0.848072048
SD	0.360845925	0.589929961	0.431407123

	PBMCs + A375 (24H)	PBMCs + A375 (48H)	PBMCs + A375 (72H)
	0.453449845	3.087611	1.442580101
	1.311701	0.712669	0.60828
	1.153246011	0.656571	0.004361528
	0.201503063	1.758728	0.013133598
	0.171679174	2.578059759	0.219525309
Mean	0.658315819	1.758727752	0.457576107
SD	0.538361235	1.089291518	0.602607939

	PBMCs + MEL28 (24H)	PBMCs + MEL28 (48H)	PBMCs + MEL28 (72H)
	0.334442198	3.313915893	1.116616
	0.794146477	2.33952516	1.334524471
	0.604956254	1.641239	0.898707542
	0.617599374	0.741583059	0.466011882
	0.487210236	0.169932039	0.293761013
Mean	0.567670908	1.64123903	0.821924181
SD	0.170325998	1.250957287	0.436213224

Coculture Model: CSNK1A1

	PBMCs control (24H)	PBMCs control (48H)	PBMCs control (72H)
	0.523488595	1.080034005	0.912982079
	0.732542604	0.790507418	1.457181766
	1.386920332	1.550966632	1.252852587
	1.090534549	0.954336453	0.62949724
	1.724125772	0.791320931	0.953081884
Mean	1.091522371	1.033433088	1.041119111
SD	0.484504086	0.313807294	0.320757469

	PBMCs + A375 (24H)	PBMCs + A375 (48H)	PBMCs + A375 (72H)
	0.306325173	0.533049291	0.225880613
	1.832487465	0.423448965	0.571686016
	1.434461211	1.028767395	0.651057942
	0.466593845	0.927531658	1.178963204
	0.418391336	0.65634621	0.684420856
Mean	0.891651806	0.713828704	0.662401726
SD	0.694094878	0.257469007	0.341404931

	PBMCs + MEL28 (24H)	PBMCs + MEL28 (48H)	PBMCs + MEL28 (72H)
	0.364575566	1.108298	1.168685158
	0.250196273	0.494471637	0.247848121
	0.75218935	1.722124	0.434791469
	0.859956998	1.505052392	0.651193093
	1.056549382	0.207986312	0.673786339
Mean	0.656693514	1.007586468	0.635260836
SD	0.339445547	0.646302229	0.345165227

Coculture Model: ILF3

	PBMCs control (24H)	PBMCs control (48H)	PBMCs control (72H)
	0.76819344	0.549947323	0.968922309
	0.164059872	0.216401496	2.968909916
	1.123493688	1.876718	0.525513127
	2.768365779	1.650968252	0.633347263
	2.55113199	1.213333	1.044452172
Mean	1.475048954	1.101473614	1.228228957
SD	1.137162181	0.707635966	0.997331165

	PBMCs + A375 (24H)	PBMCs + A375 (48H)	PBMCs + A375 (72H)
	0.631481929	0.322996	0.014510531
	0.58099089	0.855131	1.129270125
	1.007837662	0.519608	0.031377476
	0.156953136	0.079285	0.00161612
	1.047162133	0.675592	0.304021258
Mean	0.68488515	0.4905224	0.296159102
SD	0.363284004	0.302166119	0.482267194

	PBMCs + MEL28 (24H)	PBMCs + MEL28 (48H)	PBMCs + MEL28 (72H)
	0.235961361	1.071038594	1.576356348
	0.115118502	0.280888573	0.652974
	1.258278955	0.78518159	0.371286976
	2.172356936	0.929697068	0.934660212
	1.817121218	0.000617777	0.850235482
Mean	1.119767395	0.61348472	0.877102603
SD	0.922478326	0.454161782	0.446954584

Other cancers

	Normal	Colorectal cancer	Cholangiocarcinoma
	2.273917181	0.655043722	1.799115173
	2.556236063	0.320736455	1.806642375
	0.06364886	0.552964693	2.552186178
	1.217345843	0.443190149	0.22078563
	1.556634886	0.356310522	2.175651
Mean	**1.533556567**	**0.465649108**	**1.710876071**
SD	0.981727774	0.138702325	0.888980495

	Breast cancer	Gastric cancer	Thyroid cancer
	2.803136781	1.885369853	0.3189313
	3.107718579	0.642622152	1.001695805
	4.097700988	1.938634892	2.342714384
	2.003563779	2.940131612	0.41892463
	3.602709783	1.502951349	0.362128051
Mean	3.122965982	1.781941971	0.888878834
SD	0.796217141	0.829538827	0.858709644

	Ovarian cancer	Liver cancer	Melamoma
	0.377533405	0.733193709	5.516724267
	2.342714384	0.83198963	4.724213474
	3.856875854	0.814949023	4.675987992
	0.984721841	0.385775828	2.01231719
	1.447149336	0.800905484	1.996196051
Mean	1.801798964	0.713362735	3.785087795
SD	1.354423704	0.186923979	1.659598925
